# Non-Muscular Invasive Bladder Cancer: Re-envisioning Therapeutic Journey from Traditional to Regenerative Interventions

**DOI:** 10.14336/AD.2020.1109

**Published:** 2021-06-01

**Authors:** Kuan-Wei Shih, Wei-Chieh Chen, Ching-Hsin Chang, Ting-En Tai, Jeng-Cheng Wu, Andy C. Huang, Ming-Che Liu

**Affiliations:** ^1^Department of Urology, Taipei Medical University Hospital, Taipei 11031, Taiwan.; ^2^Graduate Institute of Clinical Medicine, School of Medicine, College of Medicine, Taipei Medical University, Taipei 11031, Taiwan.; ^3^TMU Research Center of Urology and Kidney, Taipei Medical University, Taipei 11031, Taiwan.; ^4^Institute of Microbiology and Immunology, National Yang-Ming University, Taipei 11031, Taiwan.; ^5^Department of Education, Taipei Medical University Hospital, Taipei 11031, Taiwan.; ^6^Department of Urology, School of Medicine, College of Medicine, Taipei Medical University, Taipei 11031, Taiwan.; ^7^Clinical Research Center, Taipei Medical University Hospital, Taipei 11031, Taiwan.; ^8^Institute of Traditional Medicine, School of Medicine, National Yang-Ming University, Taipei,11221, Taiwan.; ^9^Department of Urology, Department of Surgery, Taipei City Hospital Ren-Ai Branch, Taipei 10629, Taiwan.; ^10^School of Dental Technology, College of Oral Medicine, Taipei Medical University, Taipei, Taiwan.

**Keywords:** NMIBC, Bladder cancer, BCG, Stem cells, PRP

## Abstract

Non-muscular invasive bladder cancer (NMIBC) is one of the most common cancer and major cause of economical and health burden in developed countries. Progression of NMIBC has been characterized as low-grade (Ta) and high grade (carcinoma in situ and T1). The current surgical intervention for NMIBC includes transurethral resection of bladder tumor; however, its recurrence still remains a challenge. The BCG-based immunotherapy is much effective against low-grade NMIBC. BCG increases the influx of T cells at bladder cancer site and inhibits proliferation of bladder cancer cells. The chemotherapy is another traditional approach to address NMIBC by supplementing BCG. Notwithstanding, these current therapeutic measures possess limited efficacy in controlling NMIBC, and do not provide comprehensive long-term relief. Hence, biomaterials and scaffolds seem an effective medium to deliver therapeutic agents for restructuring bladder post-treatment. The regenerative therapies such as stem cells and PRP have also been explored for possible solution to NMIBC. Based on above-mentioned approaches, we have comprehensively analyzed therapeutic journey from traditional to regenerative interventions for the treatment of NMIBC.

Non-muscular invasive bladder cancer (NMIBC) also known as superficial bladder cancer constitute approximately 75-80% of bladder cancer [1]. Each year, bladder cancer is diagnosed about 5,49,393 patients worldwide and according to recent estimates of National Cancer Institute of US, 80,470 new patients of bladder cancer in 2019, made it 11^th^ and 4^th^ most common cancer among women and men, respectively. According to analysis of National registry data from U.S Surveillance Epidemiology and End result (SEER) program data in 2012 suggested that from 1992 to2002, approximately 4790 patients were diagnosed with a high grade NMIBC, of which only one received recommended treatment [2]. Further analysis of SEER in 2019 on the basis of demographic factors and survivors from 1975 to 2016 represent that incidence of all stages of NMIBC has been relatively stable, where its lowest and highest rate of risk is 17.7% and 37.6%, respectively [3]. Though most of the NMIBC patients underwent various surgical procedures, treatments that aimed to down regulate the disease progression and recurrence for the life time surveillances, it is more costly and also associated with patients anxiety [4, 5]. To address therapeutic needs of NMIBC along with surgical, pharmaceutical drugs such as epirubicin, BGJ398, mitomycin C (MMC) sunitinib, enzalutamide, ethacrynic acid, and tamoxifen, the bio-molecules like BCG strain, monoclonal antibodies, vaccines, immunomodulators, gene and radiation therapy also seem promising options [6]. Further, the regenerative therapy using progenitor cells, stem cells and platelet rich plasma (PRP) have also shown potential of rejuvenating bladder post-treatment.

To promote an improved adherence to best practices for NIBMC treatment, we present here a state-of-the-art, updated review of its pathophysiology and various therapeutic strategies.

**Figure 1.**
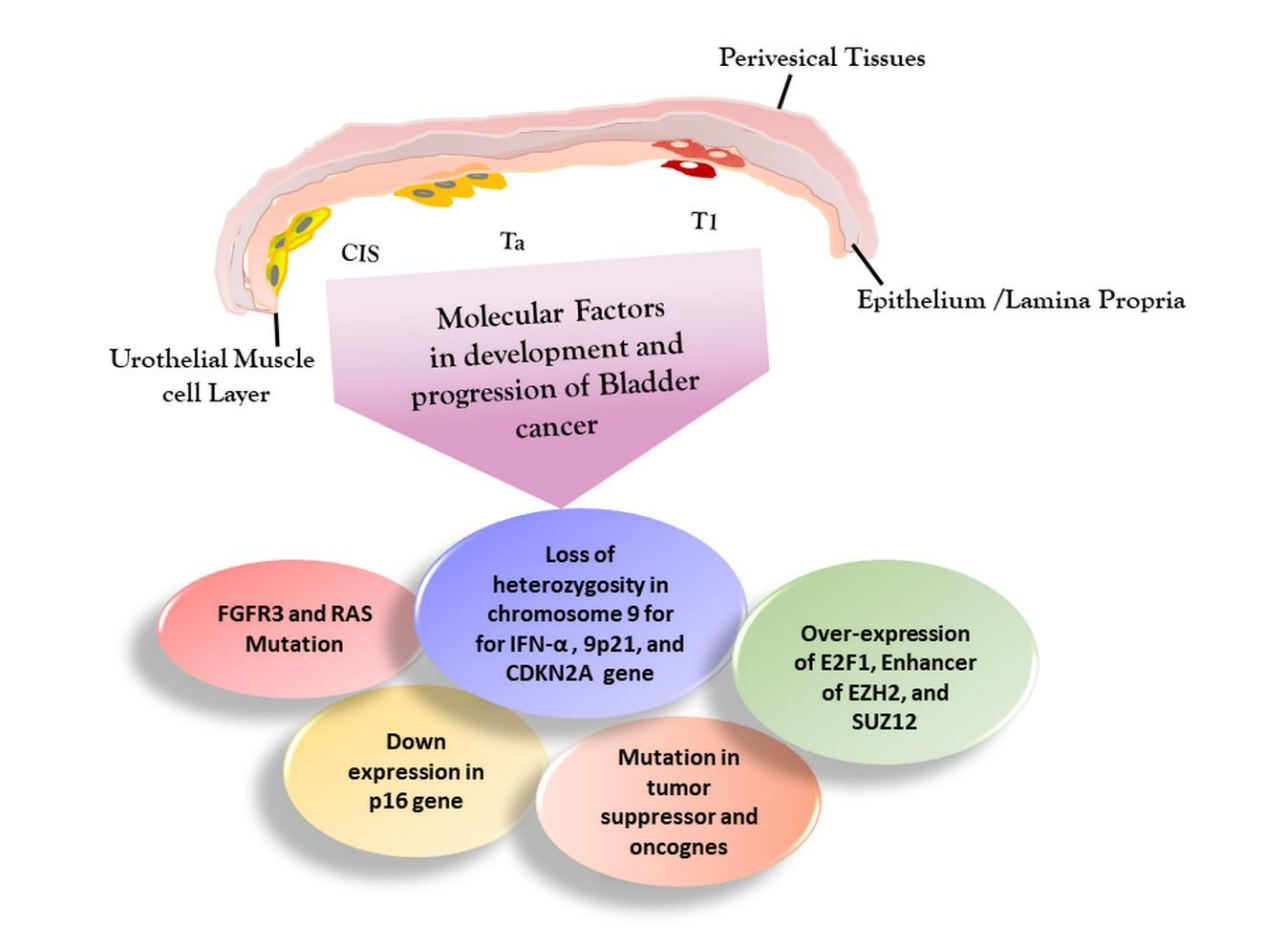
Pathologic grading of non-muscular invasive bladder cancer (NMIBC). Ta, a low-grade carcinoma is limited to epithelium/lamina propria; whereas, high grade includes carcinoma in situ (CIS) and T1, which are restricted to urothelium and muscles, respectively. Further progression of T1 leads to development of muscle invasive bladder cancer. Molecular factors such as mutated tumor suppressor, FGFR3 and RAS mutation, loss of heterozygosity in chromosome 9 for IFN-α, 9p21, and CDKN2A gene, over-expression of E2F1, Enhancer of EZH2, and SUZ12, and down expression in p16 gene are considered as major cause of bladder cancer. FGFR3: Fibroblast growth factor receptor 3, IFN-α: Interferon alpha, CDKN2A: Cyclin-dependent kinase inhibitor 2A, E2F1: E2F Transcription Factor 1, EZH2: Enhancer of zeste homolog 2.

## Non-Muscle Invasive bladder cancer (NMIBC): Burden, Pathophysiology, and Progression

NMIBC is a heterogeneous disease with high rate of recurrence and progression [7]. Its histological grade is the most important prognostic factor for determining tumor progression. In 1973, WHO proposed a grading system for papillary urothelial carcinoma on the basis of cellular atypia into three numerical categories (G1, G2, G3) [8], which was consistently criticized and modified several times. In 2004, the WHO again standardized the grading of urothelial carcinoma into three categories i.e. papillary urothelial neoplasma of low malignant potential (PUNLMP), low grade carcinoma and high grade carcinoma [9]. However, this grading system was further revised in 2016 remained more or less similar to 2004 guidelines. The cases of urinary bladder cancer (UBC) are more frequent in Unites state of America (USA), Canada and West Europe and lest reported in Asia, East Europe and Central Africa [10, 11]. However, a significant increase in UBC patients among developing countries is predicted in near future [10]. Around 60% of urothelial cancers are NMIBC with 50-60% recurrence ability [12]. Around 5,50,000 new cases of UBC have been recorded alone in 2018 [13], and by 2030, Germany, France, Bulgaria and Brazil may encounter highest increase in bladder cancer cases [14]. The risk of bladder cancer and its recurrence exhaust economical resources in providing better care, treatment, observation and periodic monitoring [14, 15]. Thus, the global attention is needed to address affordable diagnostic, treatment and management of UBC and NMIBC [15]. High risk of UBC/NMIBC has been associated with aging, exposure to carcinogens such as aromatic amines, polycyclic aromatic and chlorinated hydrocarbons, stones in urinary tract, contamination of water with pollutant such as arsenic etc. and tobacco smoking along with radiotherapy and infection of *Schistosoma haematobium* [10, 15-17]. Moreover, food behavior might also affect the chance of developing the bladder cancer, as the food metabolites are often secreted out though urinary tract [16]. The reduction in use of tobacco and exposure to hazardous chemicals may significantly reduce the unexpected burden of NMIBC and UBC [18]. The high degree of bladder cancer among first- degree relatives of UBC is mainly associated with two genes, i.e. acetylator N-acetyltransferase 2 (NAT2) variants and glutathione S-transferase mu1 (GSTM1)-null genotype [17].

Based on the magnitude of tumor metastasis and invasion, NMIBC has been classified into three stages, namely Ta, T1 and carcinoma in situ (CIS) (Fig. 1). As per WHO characterization guideline 2016, all three stages of NMIBC are further sub-grouped into low and high NMIBC [19]. A number of panels from European Urological Association (EUA) and American Urological Association (AUA) have also released guidelines to provide considerable consensus to familiarize clinicians regarding the NMIBC management in their current practice (Table 1) [20]. CIS is a flat non-papillary high grade NMIBC restricted to urothelium and Ta is low grade NMIBC which reaches to epithelium or mucosa [8]. Whereas, T1 high grade NMIBC (T1HgBC) reaches to lamina propria [21]. Further, progression from high grade T1 NMIBC stage leads to 33-48% incidence of muscle invasive bladder cancer (MIBC) with 69% to 80% recurrence rate [22]. Of all NMIBC cases, only 10% belong to CIS; whereas rest 70% and 20% are Ta and T1 stage, respectively. Moreover, CIS is non-invasive and multicentric urothelial carcinoma (UC), and considered as precursor of invasive muscle bladder cancer [23, 24]. Cells of CIS are differentiated based on morphological characterization such as, nuclear membrane irregularities, growth of CIS along the basement membrane, exposed nucleoli, hyperchromasia, enlarged nucleus and architectural disarray [25]. In addition, T1HgBC is characterized as mainly papillary tumor with nodular appearances. Further, penetration in muscularis mucosae increase the risk of recurrence and progression [26]. Additionally, bladder neck inclusion, lymph vascular invasion and pyuria also increase the risk of progression and recurrence. The molecular mechanism related to NMIBC etiology and progression has not been well established. However, the loss in heterozygosity (LOH) of chromosome 9 specifically in 9p [27, 28], loss of CDKN2A, down expression in p16 along with mutation in tumor suppressor genes and oncogenes have been associated with NMIBC.

## Cancer stem cells (CSCs) in NMIBC and therapeutic challenges

**Figure 2.**
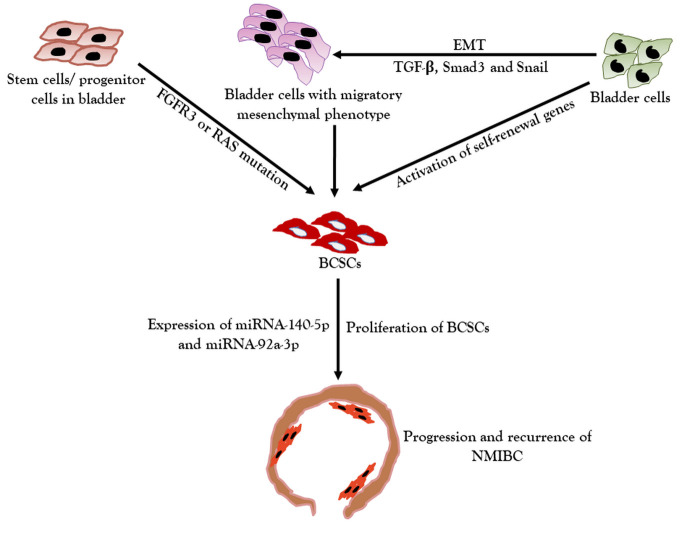
Etiology of development and progression of NMIBC. EMT is promoted through TGF-β, Smad 3 and Snail leading to transition of bladder cells into mesenchymal phenotype, which finally transform into BCSCs. Additionally, FGFR3 or RAS mutation in bladder stem cells/ progenitor cells may also triggers the transformation of stem/progenitor cells into BCSCs. Similarly, reactivation of self-renewal genes in bladder cells also transforms these cells into BCSCs, which rapidly divide and develop into bladder tumor due to dysregulated miRNA-140-5p and miRNA-92a-3p. EMT: Epithelial mesenchymal transition, BCSCs: Bladder cancer stem cells, FGFR3: Fibroblast growth factor receptor 3.

CSCs are classified as sub-category of tumor cells which have potential to proliferate and maintain heterogeneous cancer cell population in tumor [29, 30]. These cells are also capable to provide resistance to tumors from chemo- and radio-therapies [30-32]. Bladder CSCs (BCSCs) in particular, have been associated with recurrence, heterogeneity, chemo-resistance and metastasis of bladder cancer [33]. BCSCs can originate from both mutations of progenitor or stem cells and activation of self-renewal genes in dedifferentiated cells (Fig. 2). Similar to stem cells, signaling pathways for proliferation and differentiation including Sonic Hedgehog (SHh), Notch, Janus kinase/signal transducer and activator of transcription (JAK/STAT): STAT3 activation and Wnt signaling pathways are associated with BCSCs [33-35]. In transgenic mice model the FGFR3 or RAS mutation leading to development BCSCs and eventually the NMIBC has been reported [36].

In a clinical study, NMIBC samples rich in CSCs were found to express higher levels of ABCG2, and suggested to target MAPK/ERK pathways to inhibit tumor progression and recurrence [37]. Other stimulatory effects such as hypoxia dedifferentiation and epithelial mesenchymal transformation (EMT) promote transformation of bladder cancer cells into BCSCs [29]. BCSCs being resistant to conventional therapeutic approaches pose risk to recurrence, resistance and progression of NMIBC [38]. Chemo-resistance among recurrent bladder cancer is more prominent compared to first generation bladder cancer [32], which hampers to in-depth accessibility of drug to tumors [39]. By means of anti-resistance characteristics, BCSCs pump out anti-tumor drugs metabolism of drugs, and inhibit anti-apoptotic activity through down-regulation of immunomodulatory response to mitigate tumor [33]. It has been well-established that the expression profile of micro RNAs (miRNAs) such as miRNA-21, 22, 24 134, 143, 145, 296, and 470) play a crucial role in maintaining pluripotent nature of embryonic stem cells (ESCs) through self-renewal, proliferation and differentiation potential [40-42]. While exploring the role miRNA in CSCs, it has been reported that mi-RNA mediates cancer stem cells pluripotency through TGF-β pathways [39]. In addition, TGF-β in combination with Smad 3 and Snail also promotes EMT [43].

**Figure 3.**
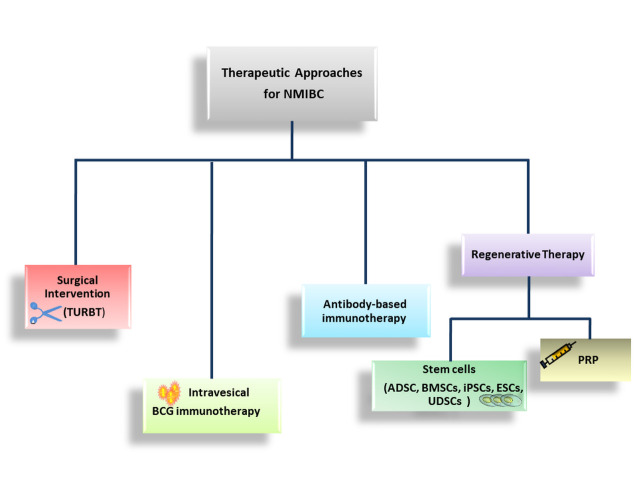
Therapeutic measures for NMIBC, which includes intravesical chemotherapy, BCG immunotherapy and surgical intervention mainly TURBT. Besides, the advanced regenerative therapeutic options include such stem cells, PRP and other biomaterials. ADSCs: Adipose-derived stem cells, BMSCs: Bone marrow stem cells, iPSCs: Induced pluripotent stem cells; ESCs: Embryonic stem cells, UDSCs: Urine-derived stem cells, and PRP: platelet-rich plasma.

Furthermore, the overexpression of E2F transcription factor 1 (*E2F1)*, Enhancer of Zeste Homolog 2 (*EZH2)*, and *SUZ12* genes have been correlated with progression, migration and invasiveness of cancer. Nevertheless, the anticancer drugs acting on cell lines over-expressing these genes promote the expression of CSCs related genes including *CD44, KLF4, OCT4, and ABCG*. The *EZH2* gene has been demonstrated to functionally silence tumor suppressor gene and miRNA along with activation of oncogenic signaling pathways [44, 45]. *EZH2* gene in particular, silences pro-apoptotic miR-31 and miR-205 which results in increased anti-apoptotic activities in bladder tumor [46]. Therefore, over-expressing these anti-apoptotic genes have been targeted both for diagnostic and treatment purposes. The attempts have already made to downregulate the expression of *EZH2* gene through miRNA 26a resulting in increased apoptosis and inhibited cancer cell proliferation [47]. The direct and indirect down-regulation of *EZH2* gene have been achieved through pre-clinical drugs such as DZNEP, D9, EPZ-6438, SAH-EZH2 peptide, NSC745885, gambonic acid and methyl jasmonate [48]. Of note, the miRNA-based techniques could be more efficacious in monitoring progression of NMIBC. This was evident through expression profile of miRNA-140-5p and miRNA-92a-3p in urine cell sample indicating these molecules as progressive tumor markers [49]. In a clinical study, it was observed that the urothelial stem cell (USC) markers, ΔNp63, integrin β4, CD47, and CD44v6 were over-expressed in trigone and posterior wall of bladder tumor and associated with poor recurrent-free survival in tumor patients [50]. Even our research group demonstrated that ovatodiolide, a macrocyclic bioactive component isolated and purified from *Anisomeles indica* inhibited M2-polarized tumor-associated macrophages (M2 TAM) polarization, suppressed EV cargos derived from M2 TAMs, leading to repressed β-catenin/mTOR/CDK6 signaling. These outcomes pre-clinically evidenced that ovatodiolide may act as a single or adjuvant agent for drug-resistant bladder cancer therapy [51].Thus, the BCSCs could be targeted to develop diagnostic and therapeutic approach to inhibit the relapse of NMIBC [52]. However, population heterogeneity in BCSCs remains a major challenge due to difficulty in differentiating BCSCs, normal cells, bladder progenitor and stem cells. In the next sections, we have described in detail about the various treatment approaches towards NMIBC (Fig. 3).

## Surgical management of NMIBC

### Transurethral resection of bladder tumor (TURBT)

**Figure 4.**
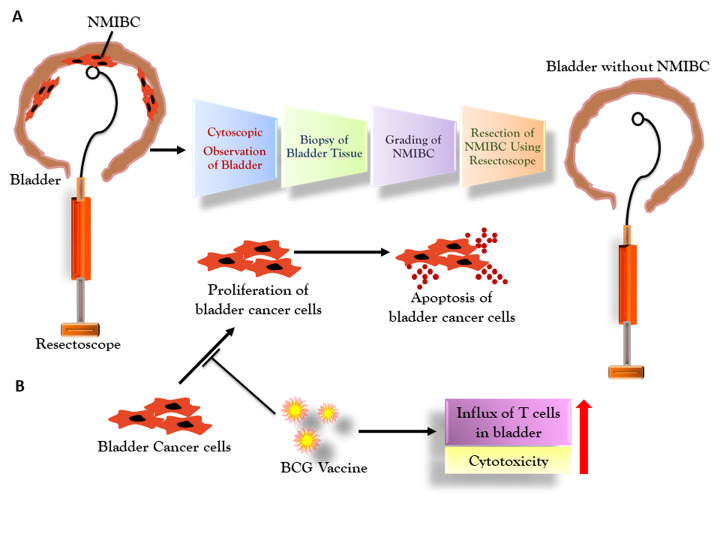
Surgical and immunotherapeutic approaches for NMIBC. (A) Transurethral resection of bladder tumor (TURBT) for NMIBC. The bladder tumor is surgically removed, and presence of detrusor muscle is an indicator of effective removal. (B) Representative intravesical BCG immunotherapy, which induces influx of T cells and cellular cytotoxicity towards bladder cancer cells and promote their apoptosis.

TURBT is standard surgical therapeutic procedures to remove cancer lesions from NMIBC patients (Fig. 4A) [53]. During this process, tumor is initially located to identify its grade through cytoscopy. Thereafter, fresh urine is analyzed for detecting bladder tumor grade and multiple markers [54-56]. TURBT is employed to eliminate all possible tumors, which are examined further in-depth to determine its nature [56]. However, this therapy might cause haematuria, bleeding, irritation and clinical bladder perforation [57]. The en-bloc TURBT is an advanced resection technique to decrease the recurrence rate of bladder tumor [58]. The presence of detrusor muscle in resection tissue sample is a good indicator of successful of TURBT [59]. A meta-analysis reported that en-bloc TURBT reduced operation and catheterization time, and hence the hospitalization stay [60]. It further reduced post-operative side effects along with decreased recurrence rate. The assistance with imaging methods such white light and narrow band imaging also inhibit the recurrence of NMIBC post-TURBT [61]. It has been noted that clear identification of flat and small lesions such as CIS is really hard, which may be overcome by artificial intelligence through its ability to analyze various tumor cystoscopic images [62, 63]. The study indicated the convolutional neural network and deep learning techniques can be helpful to diagnosing NMBIC. Moreover, the efficacy, recurrence and safety of TURBT mostly depend on expertise of surgeon, as results, the exhaustive experience of surgeon significantly reduce the risk of recurrence with improved diagnosis of NMIBC [64, 65]. It has been reported that the recurrence rate was high among junior surgeon and independent of detrusor muscle presence in the specimen [66]. The relapse of NMIBC is frequent after first TURBT among Ta and T1 patients; thus, repeated TURBT is necessary to control progression and clearance of NMIBC [67].

## Immunotherapeutic approaches towards NMIBC

Immunotherapy stimulate patient’s immune response with help of exogenous cytokines and vaccines to improve its capacity against specific tumor associated agents, and eliminate tumor [68]. It represents viable treatment strategy as it attains a direct contact between therapeutic agent and tumor via intravesicles, and respond through cystoscopic surveillance [69].

## Intravesical Immunotherapy with BCG

Intravesical BCG most commonly used and established immunotherapy for NMIBC (Figure 4B). According to American urological association (AUA) guidelines, the NMIBC patients of s recurrent low-grade (Ta tumors) high-grade (T1) and carcinoma in situ (CIS) are highly recommended to undergo BCG immunotherapy [70]. BCG being a stable, safe, effective, economic, and multi-route vaccine along with its ability to express other antigen is widely used as a potent candidate to address therapeutic issues for NMIBC [71-73].BCG strain used for NMIBC immunotherapy is developed from *Mycobacterium bovis* [74]. The efficacy of BCG varies according to strain variant, and it seems that the late Russian and Connaught BCG strains were most effectual in inhibiting proliferation of T24 and J82 tumor bladder cell lines through inducing cytokine production [75]. Though the mode of BCG action to overcome bladder cancer is not well-established [76], the major clinical effect is correlated with its potential to trigger immune response, and resultant increase in cytokine production, influx of granulocytes and mononuclear cells in bladder, and eventually the inhibition of cell proliferation and promotion of apoptosis in bladder tumor [77-81]. It is also considered that along with specific immune response, innate immunity play a crucial role in providing immunity through BCG vaccination [82]. In a seminal report, BCG S4-Jena strain have been demonstrated to inhibit cell proliferation and promoted apoptosis in Cal29 and T24 bladder tumour cell line, implying high potential of BCG strain against NMIBC [76]. While *in-vitro* testing, the BCG sub-strains i.e. Moreau, Tice and RIVM, have also shown inhibitory effect on human bladder cancer cell line, T24, which has been attributed to presence of cytotoxic mycolic acids on cell surface of these strains [83]. Moreover, presence of Th1 cytokines such as TNF-α and IFN-γ enhance tumor cell proliferation by inhibiting activity of BCG strains. Nonetheless, BCG when co-cultured with bladder cancer cell lines T24 and J82, showed increased cancer cell apoptosis through reducing cellular telomerase activity [81]. Notably, an interesting study reported no inhibitory effect of cell proliferation of T24 by BCG alone; however only BCG-infected dendritic cells-mediated inhibition was found to be associated with ability of cells to secrete IL-12 and TNF-α [84]. Moreover, the administration of BCG treated MBT-2 tumor cells in murine lymph node improved the secretion of IL-2 and IFN-γ from murine lymphocytes. The risk of recurrence even after BCG treatment in NMIBC patients is clinically correlated with high secretion of IL-8 by lymphocytes [85]. Interestingly, a combined treatment of BCG and interferon (IFN) has revealed a synergistic effect on the immune response, and this intervention with IFN reduced BCG dosage in order to suppress toxicity [86]. However, results of random clinical trials are not much supportive for this combinatorial therapy.

It has also been reported that IL-10 could inhibit cytotoxic behavior of BCG on NMIBC [87], which could be further improved by blocking its secretion leading to improved TNF-α content. In a clinical study, urine samples of NMIBC patients after BCG instillations was found with elevated levels of cytokines, chemokines and C-X-C motif chemokine 10 (CXCL10 or interferon-inducible protein 10: IP10). The CXCL10 plays a crucial role in recruitment of immune effector cells [88]. Additionally, frequency of instillation of BCG also impact response during treatment of NMIBC. In particular, repeated BCG administration helps to increase the robust influx of T cells in bladder tumor inhibits relapse of NMIBC [89]. On contrary, in a long-term clinical trial (3 years), no significant improvement in therapeutic efficacy, inhibition of recurrence and progression of tumor was observed by BCG treatment in NMIBC patients [90]. Instead, the long-term BCG therapy also led to severe adverse events. However, the attempts have been carried out to improve the therapeutic efficacy by combining BCG with other drugs. A randomized clinical trial showed that though mitomycin C improved therapeutic efficacy of BCG, it also increased toxicity among mid and high risk NMIBC patients, which mitigate its chance to be further explored as a safe intervention [91]. In a murine model, acetylic salicylic acid and pentoxifylline seem to be effective in improving BCG efficacy [92]. In a systemic meta-analysis, it was concluded that compared to BCG alone, the synergistic effect of intravesical BCG and chemotherapy improved recurrence free survival, overall survival and disease specific survival with significantly decreased rate of fever, irritative symptoms and hematuria therapy among NMIBC patients [93]. Nonetheless, the therapeutic efficacy of BCG is undermined owing to its associated local and systemic side effects such as dysuria, haematuria, cystitis, sepsis, malaise and fever [83, 90]. Moreover, in case of observed severe side effect such as sepsis, the BCG therapy is withdrawn and followed by tuberculosis treatment [94]. To overcome limitations of BCG and increase its efficacy, recombinant modification of BCG strains has been attempted to express immunomodulatory component on its surface [82]. A seminal study on murine bladder tumor showed that the recombinant poly BCG vaccine instillation increased the influx of immune cells in bladder tumor, with elevated cell apoptosis eventually leading to the survival of mice [95]. The expression of murine interleukin-2 (mIL-2) on the surface of BCG improved therapeutic efficacy and rBCG cytotoxicity [96, 97].Telomerase reverse transcriptase gene promoter (*TERTp)* mutations and TERT hypermethylated oncological region (THOR) are considerable genetic change which influence telomerase activity and proliferation and progression of NMIBC [98]. The point mutation (c.1-146G>A) in *TERTp* resulted in recurrence free survival among NMIBC patients [99].

## Other recent advances of immunotherapy

The unique structure and microenvironment of bladder provides opportunity to bladder tumor to bypass immune response and immunological therapy [100]. The unique balanced immune response to self-antigen, microbial and foreign antigen in bladder provides opportunity to develop immunotherapy for NMIBC. The bladder curtail immune response against self-antigen by secreting anti-microbial agents to neutralize infectious and microbial agents [101]. Bladder tumor also neutralizes tumor infiltrating lymphocytes (TILs), increase IL-10 release, accruement of immunosuppressive myeloid cells and regulatory T cells (Tregs) [102, 103]. Bladder cancer cells express immune ligands such as programmed death-1 receptor (PD-1), PD-L1, lymphocyte-activation gene-3 (LAG-3), TIM-3 and cytotoxic T-lymphocyte antigen-4 (CTLA-4) on its surface to evade immunological response and checkpoints [100, 104]. Earlier preclinical studies have suggested that CTLA-4 and PD-1 could be used to develop antibody-based therapy to overcome tumour cells potential to inhibit immune checkpoints [104, 105]. Further, expression of CD-40 antigen on surface of bladder tumor cells also trigger the influx of TIL and immune cells in NMIBC resulting in increase in apoptosis of tumor cells, This indicate that development of anti-CD40 based test to target bladder cancer [106]. Similarly, Newton et al. developed monoclonal Ab, Anti-IL-10R1 which demonstrated systemic therapeutic effects in murine bladder cancer metastasis model, and hence may prove useful in clinical practice for treating bladder cancer in high-risk patients [107]. In another attempt, an immunocytokine,NHS-muIL12, consisting of two molecules of IL-12 combined with NHS76, a tumor necrosis-targeting human IgG1, reduced the myeloid derived suppressor cells, tumor related TGF-β and macrophages in tumour microenvironment resulting in reactivation of Th cells to activate immune response against MB49^luc^ bladder tumors in mice [108]. Monoclonal antibody Atezolizumab has also been considerably effective and safe in treatment of bladder cancer patients [109]. This therapy interferes with PD-1L/PD-1 checkpoint and inhibit the immunosuppressive property of bladder tumor cells. Interestingly, the therapeutic efficacy of NHS-mul12 in preclinical model was improved when applied synergistically with anti-PD-L1 antibody, which was revealed through promoted the T cell influx, its activation and function within tumor cell microenvironment [110, 111].

## Future prospects of regenerative therapy for NMIBC

Though immunotherapy, chemotherapy and cystectomy in case of high-risk NMIBC are used to reduce the progression and recurrence, the tissue regeneration still remains a challenge. Hence, regenerative therapy seems to possess high potential in regenerating bladder wall tissues after the prognosis and treatment of NMIBC. Tissue engineering provide a two major reconstruction approaches i.e. acellular and cellular to regenerate neo-tissues that can replace damaged or resected tissues due to NMIBC [112].

**Figure 5.**
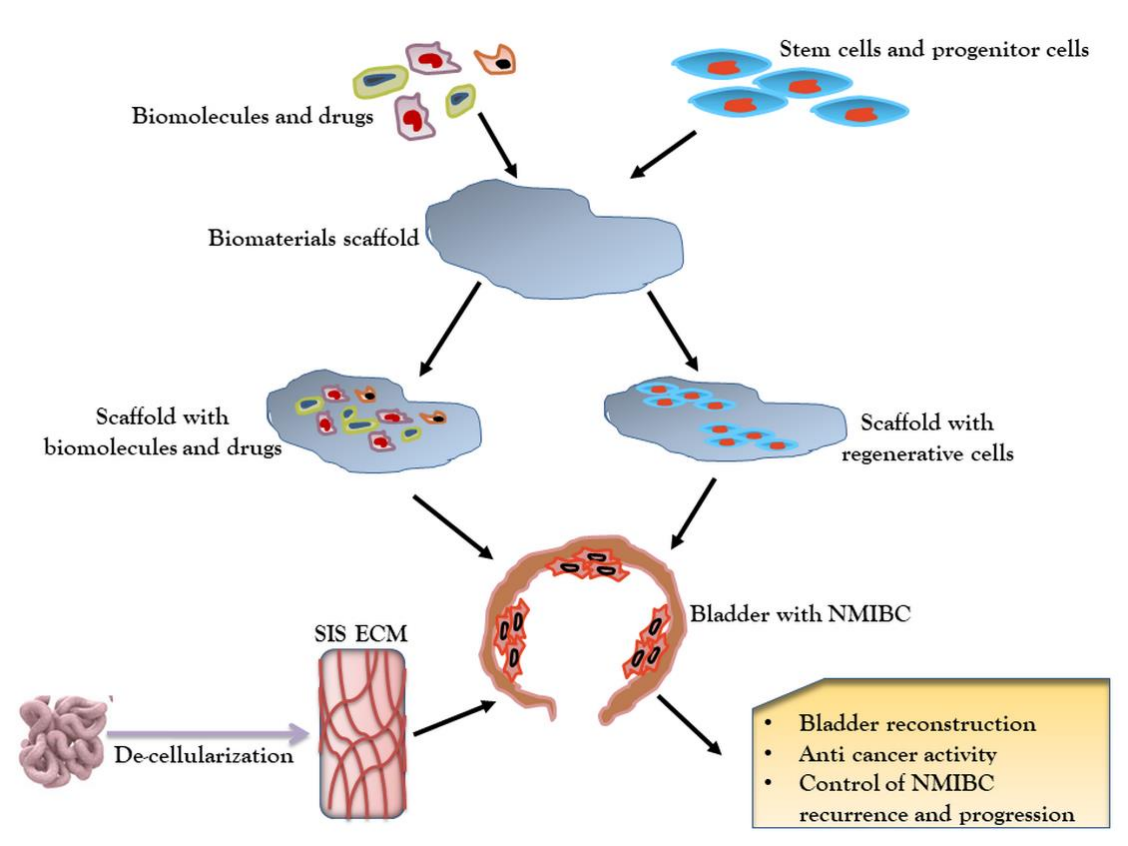
Regenerative approaches using biomaterials. Bio-molecules, drugs, stem cells and progenitor cells are entrapped in scaffold and could be used to deliver therapeutic impact at target sites. Further, small intestine is de-cellularized to produce SIS-ECM, a biomaterial for treatment of NMIBC. SIS-ECM: Small intestinal submucosa extracellular matrix.

## Biomaterials and Scaffolds in regenerative therapy of NMIBCs

Congenital and progressive bladder disorders lead to bladder damages, and the current therapies seems difficult to provide complete and long-lasting therapeutic relief [113, 114]. Moreover, adverse effects such as infection, urine disorder, stone development, metabolic complexity and recurrence of disease limits therapeutic application of conventional approaches. In recent years, the remarkable achievements in tissue engineering provided the opportunities to develop cell/cell-free and biomaterials-based therapeutic alternatives (Fig. 5).

The biomaterials such as hydrogels are widely used to deliver regenerative cells and cell secretome to address the necessity of regenerative therapy. The biocompatible biomaterials provides structural support, micro-environment for regeneration and protective role from immune response [115-118]. Moreover, the biomaterials for bladder therapy need to be biodegradable, blockade to urine seepage, easy to design in hollow and spherical tube, promote essential diffusion of oxygen and nutrients [117]. Reconstructing bladder and urinary tract post-surgical intervention in bladder cancer patients is crucial [119] and both seeded and non-seeded biomaterials seems prospective candidates. A magnetized chitosan-based scaffold injected improved retention time of BCG, strengthened immune response and enhanced anti-tumour activity in rat model of superficial bladder cancer [120]. The synergistic application of interleukin 12 (IL-12) and chitosan was also able to control orthotopic bladder tumor in mice [121]. This IL-12 seeded chitosan increases the influx of macrophages, granulocytes and effector memory cells along with controlled influx of myeloid-derived suppressor cells (MDSCs). Silk fibroin has been preclinically tested in murine model for bladder augmentation. In a report, the engrafted silk tube promoted regeneration of urothelial and SMCs leading to reconstructed the bladder wall without any major adverse event [122]. Similarly, spider silk from *Nephila edulis* seems to be a prominent biomaterial candidate, as it maintains *in vitro* survival, adhesion and proliferation of human urothelial cells [123]. During *in vitro*, nanoscaffold of poly(lactic-co-glycolic acid) (PLGA) has been demonstrated to improve the proliferation and adhesion properties of human bladder smooth muscle cells [124]. *In vitro* implantation of polyglycolic-acid scaffolds seeded with autologous ADSCs and MDSCs in the bladder submucosa, suppressed fibrosis in rabbit the [125].

These widely reported preclinical and *in vitro* studies establish the futuristic role of biomaterials in regenerative treatment of NMIBC. However, most of the studies used to reconstruct bladder has been conducted in animal models such as rats and rabbits and the bladder construct of these animals are vary from human bladder, thus poses a challenge to mimic the similar therapeutic efficacy and safety of these materials to treat NMIBC [126]. Moreover, the presence of biomaterials could also induce microbial infection as it might act as adhesive surface for microbial attack in urine [127]. Therefore, more extensive and prospective preclinical and clinical studies are required to establish safety, efficacy and translation of biomaterial therapy.

## Regenerating damaged/resected tissues in NMIBC through Stem cells

Regenerative potential of stem cells has provided extensive opportunity to develop therapies for cardiovascular, neurodegenerative, muscular disorder, bone-related and metabolic disorders, which has already validated in recent pre-clinical and clinical studies. Bone marrow stem cells (BMSCs), adipose-derived stem cells (ADSCs), embryonic stem cells (ESCs), induced pluripotent stem cells (iPSCs), urine-derived stem cells (UDSCs) are the major candidate for developing stem cell-based therapy for bladder cancer and its regeneration post-treatment. The bladder repairing ability of stem cells depends upon its ability to differentiate into bladder smooth muscle cells, urothelial and endothelial cells to [128]. Conditioned by neonatal urothelial cells, human BMSCs were able to differentiate into urothelial cells, thus provides an *in vitro* evidence of reconstructing bladder mucosa [129]. BMSCs has also been stimulated to differentiate into smooth muscle cells (SMC) and urothelium-like cells in presence of SMC or SMC-conditioned medium [130]. In radiation-injured urinary bladders of rats, the implanted BMSCs restored the structural organization of SMCs and nerve fibres [131]. Further; the potential of BMSCs and progenitor cells to regenerate bladder in patients of spina bifida [132, 133], encourages the use of BMSCs to develop regenerative therapy for NMIBC. In a seminal study, localization of magnetically labelled BMSCs into rabbit bladder post-TURBT improved bladder regeneration [134]. The differentiation of BMSCs into bladder cells is induced by stimulatory conditions and co-culturing it with urothelial or SMCs. The presence of urothelial cells induces BMSCs to differentiate into urothelial-like cells [129]. The scaffold with better structural supports and porosity and hypoxia preconditioning also improves bladder regenerative potential of BMSCs [135]. In addition, seeding BMSCs on to the scaffold also exert an additive effect on its bladder regenerating potential post-TURBT. Seeding of BMSCs in thin-film scaffold of poly (1,8-octanediol-co-citrate) and its infusion in rat partially regenerated bladder [136]. This polymer is chemically modified to release vascular endothelial growth factor (VEGF), fibroblast growth factor 2 (FGF-2), and insulin-like growth factor 1 (IGF-1) to promote angiogenesis [137]. *In vivo* infusion of small intestinal submucosa (SIS) seeded with either BMSCs or SMCs in dog, promoted synthesis of smooth muscles and restored the structure of bladder [138]. Similarly, the seeding of BMSCs in SIS and its infusion in baboon indicated the augmented structural and functional bladder recovery [139]. The genetic modification of MSCs also showed that over expression of Cyr61 increases bladder SMCs, vessel number and peripheral nerve cells resulting in functional and structural regeneration of rat bladder *in vivo*. Moreover, micro RNA 9-3p (miR 9-3p) released from exosomes of BMSCs, down regulates endothelial specific molecule-1 present on bladder cancer. This results in reduced cancer cell viability, invasion, mobility and increased bladder cancer cell apoptosis [140].

ADSCs are another widely studied source for regenerative therapy and provide opportunity to evaluate its application in NMIBC treatment. ADSC and ADSCs-conditioned medium inhibits the bladder cancer cell line T24, and EJ in S phase through downregulating and up regulating the expression of CDK 1 and cyclin A, respectively [141]. This regulation of bladder cancer cell progression has been found associated with control of caspase3/7 and Wnt/β-catenin signaling pathways. Bladder acellular matrix (BAM) loaded with human ADSCs exerts synergistic effect on bladder and smooth muscle regeneration, angiogenesis with improved in bladder volume in canine model [142]. Further, though the implantation of ADSCs into porcine model had resulted in partially regenerated bladder, only small fraction of ADSCs survived and found differentiated [143]. These preclinical and *in vitro* studies indicate that ADSCs could be a potential candidate for bladder regeneration post TURBT for NMIBC treatment. In rabbit model, BAM seeded with epithelial-differentiated rabbit adipose-derived stem cells revealed amelioration of graft contracture and recovery of urethral continuity; therefore, could serve as a potential substitute of urothelium [144]. However, there are various other studies which have indicated the ADSCs-associated increased risk of bladder tumor. The exposure of ADSC-conditioned media to bladder T24 cells inhibited its growth, yet imparted an increased chemo resistance to ciprofloxacin to cancer cells [145]. A conflicting result on positive effect of ADSC and conditioned medium on viability of bladder cancer cell lines, 5637 and HT-1376have been reported. This viability has been associated with the release of factors such as IL-6, IL-8, GM-CSF, MCP-1 and RANTES which are known to enhance invasiveness and mobility of bladder cancer cells [146]. In general, in spite of positive effects, the application of ADSCs is limited by its catalytic effect on cancer cells survival and imparting drug resistance in bladder cancer.

Urine derived stem cells (USCs) and progenitor cells are found in proximity of bladder, thus theoretically these cells have better potential to differentiate into SMCs and urothelial cells to regenerate bladder cells [147]. The non-invasive approach to obtain USCs, renders it an attractive source of therapeutic regenerative cells. The diabetic condition enhances the apoptosis of bladder cells and loss of its function. In an important report, infusion of USCs in diabetic rat regulated the bladder fibrosis and apoptosis of bladder SMCs resulting in its functional and contractile recovery [148]. The composite scaffold seeded with basic fibroblast growth factors and USCs regenerated bladder with newly organized smooth muscle cells, multilayered urothelial cells, consolidated submucosa region and improved bladder capacity in the rat [149]. When seeded on pig SIS scaffold, the human USCs were differentiated into SMCs and urothelial cells leading to multilayer mucosal structure identical to native urinary tract tissue [150].

These recent progress and studies have indicated that stem cells and progenitor cells could be developed as source of regenerative therapy for NMIBC. However, contradictory outcomes showing increased risk of tumor progression, invasiveness and metastasis of stem cells limits the potential of developing regenerative therapy for bladder cancer.

## Rejuvenating damaged/resected tissues in NMIBC through Platelet-Rich Plasma (PRP)

**Figure 6.**
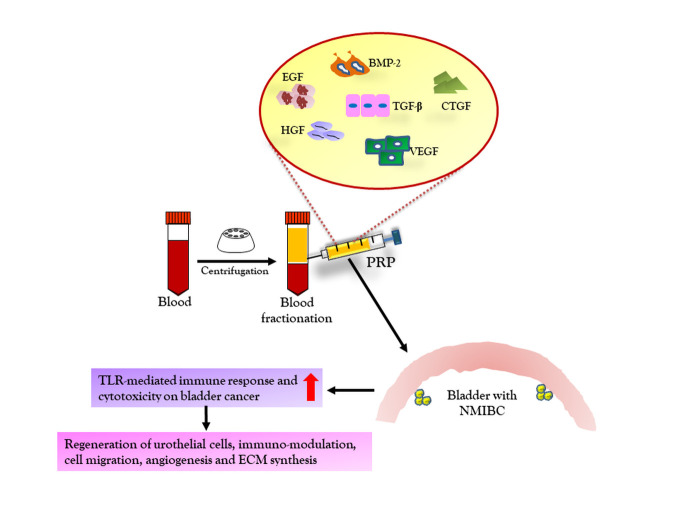
PRP-based regenerative therapy for NMIBC. PRP is autologous blood-derived platelet concentrate, which is extracted by centrifugation. PRP is a cocktail of growth factors such as TGF-β, VEGF, EGF, HGF, CTGF, BMP-2, and other bioactive components. The delivery of PRP to NMIBC target sites results in increased TLR-mediated immune response and cytotoxicity on bladder cancer. It also promotes regeneration of urothelial cells, and regulate immuno-modulation, cell migration, angiogenesis and ECM synthesis. PRP: platelet-rich plasma, TGF-β: Transforming growth factor-β, VEGF: Vascular endothelial growth factor, EGF: Epidermal growth factor, HGF: Hepatocyte growth factor, CTGF: Connective tissue growth factor, BMP-2: Bone morphogenetic protein-2, TLR: Toll-like receptor, NMIBC: Non-muscular invasive bladder cancer cells and ECM: Extracellular matrix.

PRP is harvested from blood and it is a prominent source of growth factors such as bone morphogenetic protein 2 (BMP-2), connective tissue growth factor (CTGF) epithelial growth factor (EGF), hepatocyte growth factor (HGF), platelet-derived growth factor (PDGF), vascular endothelial growth factor (VEGF), and transforming growth factor (TGF) (Fig. 6) [151]. These growth factors regulate immunomodulation, cell migration, differentiation, proliferation, angiogenesis and ECM synthesis. The PRP has already been shown to exert regenerative effect on injured or disease organs and tissues such as cardiovascular, bone, cartilage and muscles [152]. The role of PRP in regenerating urothelial cells in interstitial cystitis/ bladder pain syndrome (ICP/BPS) and recurrent bacterial cystitis has widely been reported as safety and effective [153]. In clinical trials, the repeated intravesical PRP infusion in ICP/BPS patients significantly suppressed pain and nocturia, and improved functional bladder capacity [154, 155]. In N-methyl-N-nitrosourea-induced NMIBS model of Fischer 344 rats, the PRP showed significantly better histopathological recovery with 70% decreased urothelial neoplastic lesions progression, when compared to animals receiving the same therapies administered alone [156]. These improvements in therapeutic efficacy has been suggested to be attributed to increased levels of Toll-like receptor 2 and 4 mediated immune response, resulting in elevated MyD88, TRIF, IRF3, IFN-γ immunoreactivities. Though above-mentioned evidences, implicate the potential of PRP address NMIBC therapy, the lack of pre-clinical and clinical studies limits its potential to be used as regenerative alternative. Therefore, extensive studies are needed to assess the *in vivo* and *in vitro* impact of PRP on bladder cancer and related adverse response, as it is rich in various growth factors and cytokines which may act as growth factor for matrix support to induce growth of breast cancer [157]. Taken together, current NMIBC therapeutic practices are mostly focused on TURBT and intravesical BCG immunotherapy. Though these therapies could control the progression of NMIBC, their associated side effects still limit the comprehensive therapy (Table 2). Owing to risk of recurrence and progression even after surgical intervention, finding other adequate alternatives or assistive therapeutics is urgently needed. The recent attempts focused to understand the molecular pathways and components; however, most of them are still limited to bladder cancer cell lines to find real-time effect and safety profile of the clinical trials need to be conducted at large scale. Of these, the available antibody-based immunotherapy seems most promising as these antibodies have displayed well-established clinical roles in inducing immune response against cancerous cells.

## Conclusion

NMIBC is still a major health challenge due to its progression and recurrence and the surgical facilities and expertise for TURBT limits its large-scale accessibility to address the clinical demands. Though the intravesical immuno- and chemotherapies are used as adjuvant or salvage to inhibit recurrence and progression of NMIBC, the full recovery remain inadequate. Recent progresses in regenerative therapies including stem cells and PRP also indicate the possible therapeutic opportunities due to ease of access, less complexity and long-term protection; however, further extensive studies and clinical trials are required to optimize the procedure, doses, clinical safety and efficacy for these alternatives. Notwithstanding, based on the positive outcomes of autologous regenerative alternatives, it appears that combined surgical and stem cells/PRP-based approaches might offer enhanced therapeutic efficacy.
